# Variable temperature studies of tetra­pyridine­silver(I) hexa­fluoro­phosphate and tetra­pyridine­silver(I) hexa­fluoro­anti­monate

**DOI:** 10.1107/S2056989024006972

**Published:** 2024-11-28

**Authors:** Alice G. McNelly, Kirsten E. Christensen, Amber L. Thompson

**Affiliations:** a Chemical Crystallography, Chemistry Research Laboratory, University of Oxford, Mansfield Road, Oxford, OX1 3TA, United Kingdom; Universidad de la República, Uruguay

**Keywords:** crystal structure, tetra­pyridine­silver(I) hexafluoroantimonate

## Abstract

As part of a larger study into phase transitions, the structures of tetra­pyridine­silver(I) hexa­fluoro­phosphate, [Ag(C_5_H_5_N)_4_](PF_6_) or AgPy_4_PF_6_, and tetra­pyridine­silver(I) hexa­fluoro­anti­monate, [Ag(C_5_H_5_N)_4_](SbF_6_) or AgPy_4_SbF_6_, were determined at 300 and 100 K from single-crystal X-ray diffraction. The compounds are isostructural, crystallizing in the space group *I* with *Z*′ = 1/4. Over the temperature range studied no evidence of a phase change was found. The dihedral angles between the pyridine rings are compared with similar cations from the literature and discussed.

## Abstract 2

Structures of tetra­pyridine­silver(I) hexa­fluoro­phosphate and tetra­pyridine silver(I) hexa­fluoro­anti­monate are reported from data collected at 300 K and 100 K.

## 1. Chemical context

Barluenga’s reagent, IPy_2_BF_4_, and some of its derivatives have been shown to exhibit phase transitions (Kim *et al.*, 2014 ▸; Morgan *et al.*, 2018 ▸). This study was recently extended to silver complexes of the form Ag*L*_2_*X* where *L* is a pyridine-based ligand and *X* is an anion with a single charge (Fleming, 2021 ▸; Thompson *et al.*, 2023 ▸).
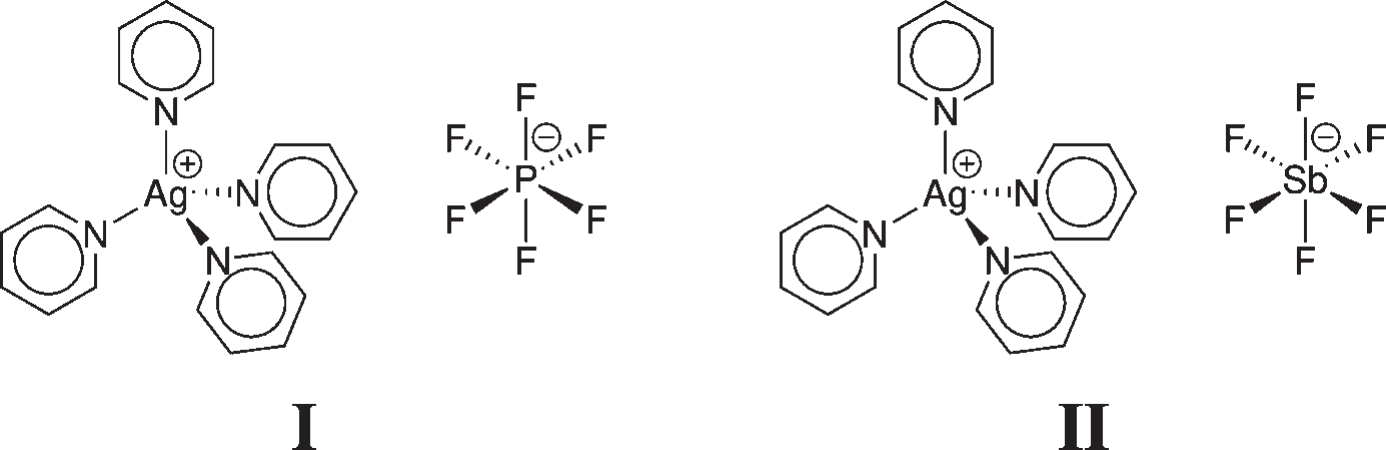


While making and studying the parent compound AgPy_2_PF_6_ and the related AgPy_2_SbF_6_, the side-products AgPy_4_PF_6_ (**I**) and AgPy_4_SbF_6_ (**II**) crystallized. Although these have not been previously reported, a number of isostructural species are known including CuPy_4_PF_6_ (Coles *et al.*, 2008 ▸), AgPy_4_ClO_4_, CuPy_4_ClO_4_ (Nilsson & Oskarsson, 1981 ▸, 1982 ▸), CuPy_4_I (Al Shamaileh & Al-Far, 2016 ▸), LiPy_4_PF_6_ (Jalil *et al.*, 2017 ▸) and LiPy_4_ClO_4_ (Harvey *et al.*, 1992 ▸). All of these crystallize in the space group *I*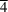 with both the cation and the anion occupying a position on a fourfold rotoinversion axis.

## 2. Structural commentary

**Figure 1:**
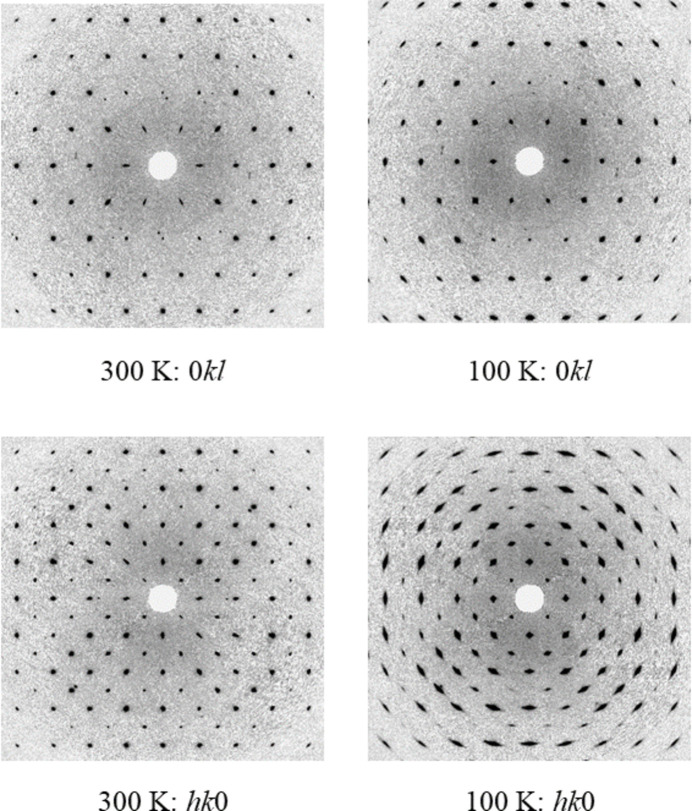
Selected reciprocal lattice sections reconstructed to 1.5 Å for the zero order layers for **I** at 300 K and 100 K.

**Figure 3:**
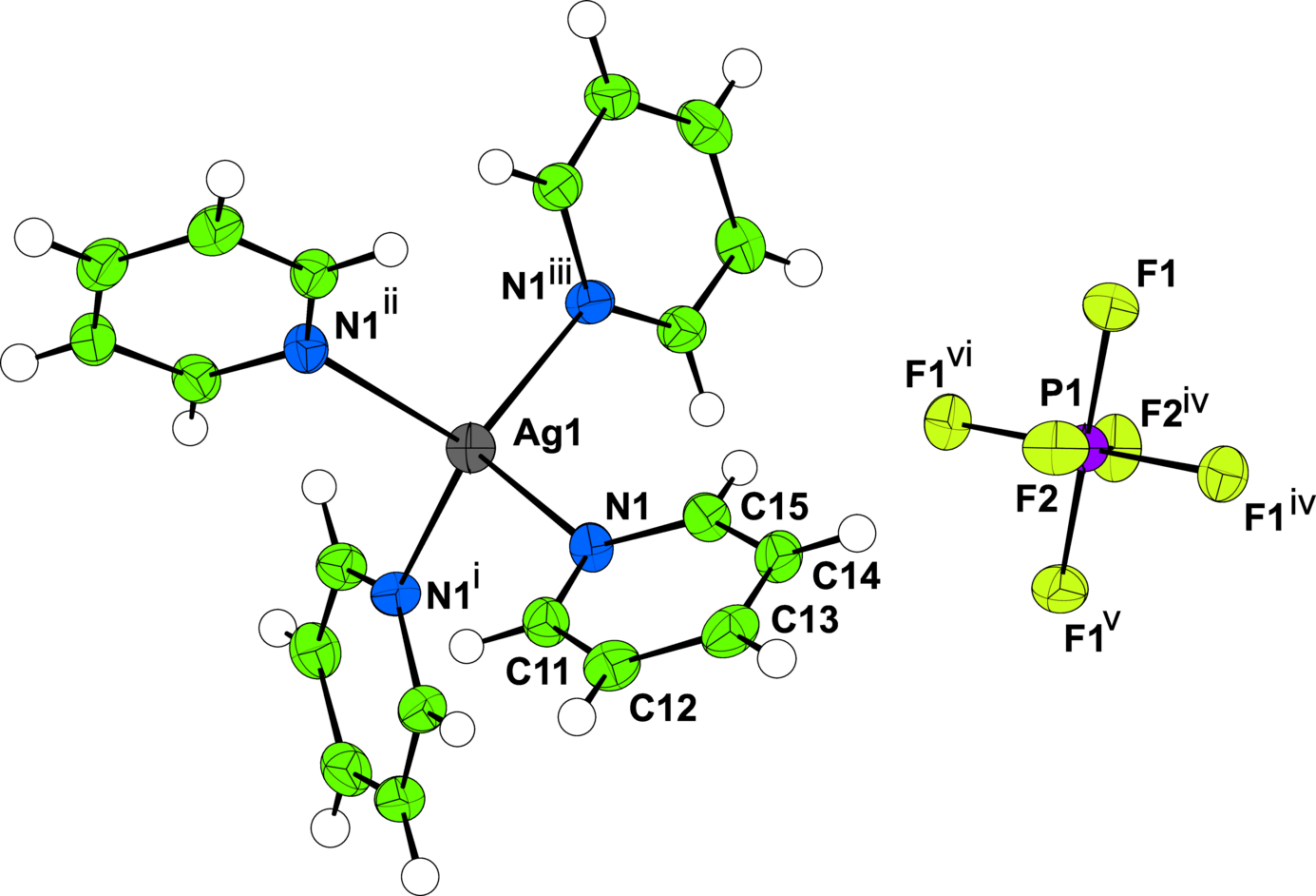
The mol­ecular structure of **I** at 100 K. Displacement ellipsoids are drawn at the 50% probability level. Symmetry codes: (i) *y*, 1 − *x*, 1 − *z*; (ii) 1 − *x*, 1 − *y*, *z*; (iii) 1 − *y*, *x*, 1 − *z*; (iv) *y* − 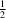, 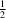 − *x*, 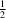 − *z*; (v) −*x*, 1 − *y*, *z*; (vi) 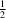 − *y*, 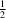 + *x*, 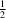 − *z*.

Single-crystal X-ray diffraction data were collected initially at 300 K, before the crystals were cooled at 200 K h^−1^ to 100 K where a similar dataset was collected. In both cases, aside from the expected unit-cell contraction and some peak broadening (presumably caused by strain), there was no evidence of a change in phase over the temperature range studied (Figs. 1 ▸ and 2 ▸). Both **I** and **II** crystallize with *Z*′ = 1/4, with the silver atom at the centre of the cation and the phospho­rus/anti­mony and one fluorine all lying on the fourfold rotoinversion axis with the other atoms on general positions (Figs. 3 ▸ and 4 ▸). In both cases, the structure forms a close-packed, body-centred arrangement of cations with the anions located in the voids. At 300 K, the voids in **I** are 118 Å^3^ contracting to 101 Å^3^ at 100 K; for **II** the void of 134 Å^3^ contracts to 120 Å^3^ showing a similar lattice contraction to **I**. Geometric parameters are given for **I** and **II** at 300 and 100 K in Tables 1 ▸–4 ▸ ▸ ▸.

## 3. Database survey

**Figure 5:**
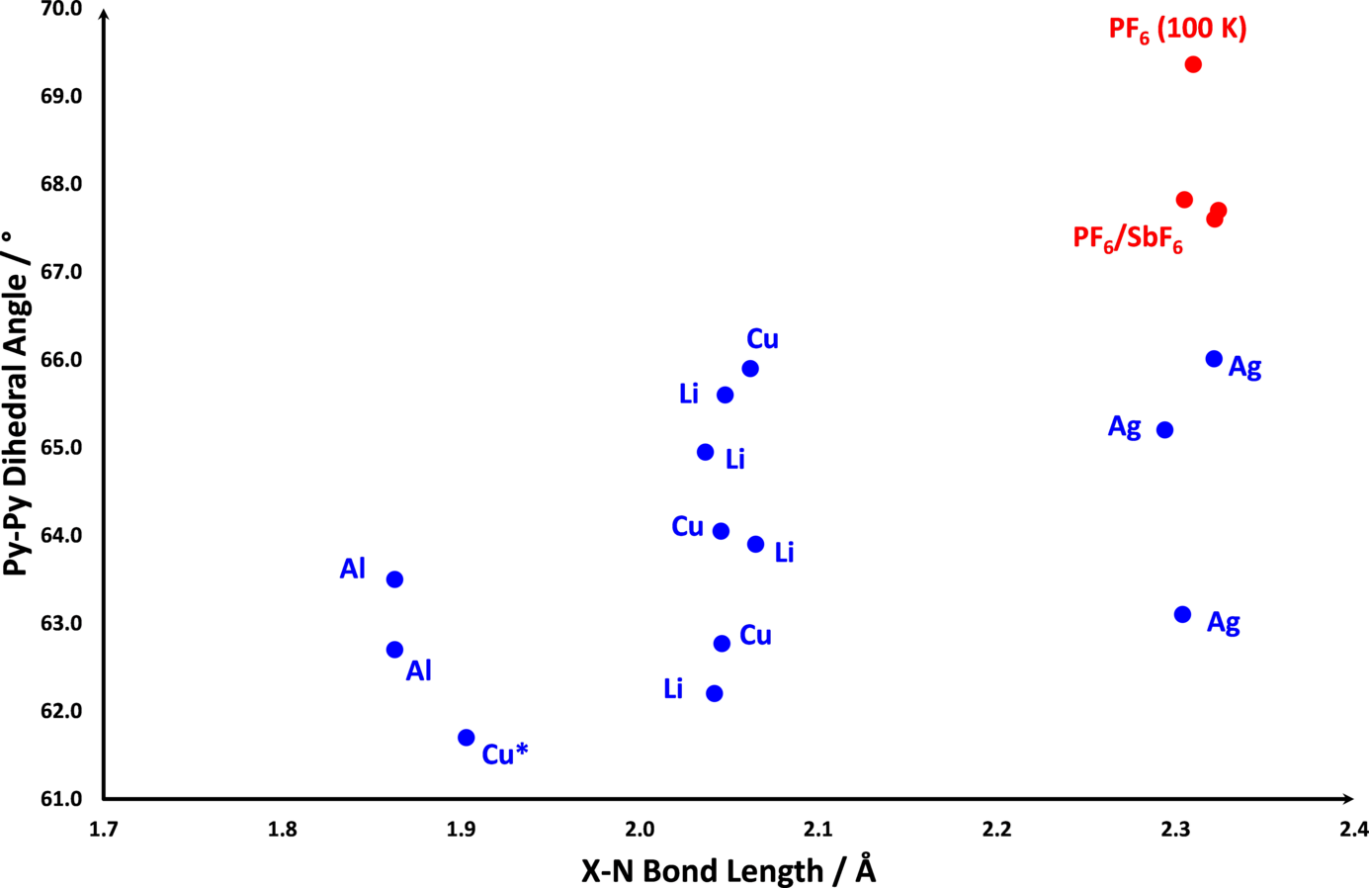
Scattergram plotting the dihedral angle between pyridine rings against the *X*—N bond distance for *X*Py_4_ cations. Data points shown in blue are from literature values taken from the CSD while those in red are from this study. Each point is labelled with *X*, and the asterisk denotes an unusually short Cu—N distance as discussed herein.

Searching the Cambridge Structural Database (CSD V. 5.45 with March 2024 and June 2024 updates; Groom *et al.*, 2016 ▸) using CONQUEST (Bruno *et al.*, 2002 ▸) for *X*Py_4_ structures in the space group *I*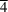 yields ten structures with three-dimensional coordinates deposited. For these, the *X*—N distance and the dihedral angles between the pyridine rings have been determined and plotted (Fig. 5 ▸). Since *X* occupies a special position, two pyridine–pyridine angles are the same and the third is related, such that as one decreases, the other increases. There is a general correlation between the *X*—N length and the dihedral angle; however, it is noticeable that the two compounds discussed herein exhibit larger dihedral angles than those previously reported and on cooling to 100 K, **I** (AgPy_4_PF_6_) shows a marked increase that is not seen for the SbF_6_ analogue.

It is also worth pointing out that the Cu—N distance for CuPy_4_I (YAGMAX, marked with an asterisk in Fig. 5 ▸; Al Shamaileh & Al-Far, 2016 ▸) is considerably shorter than the values for the other Cu structures plotted. Indeed, the value of 1.903 (4) Å is outside the inter­quartile range of 2.008–2.054 Å determined from a simple CSD search for a four-coordinate copper with four pyridine derived ligands. This structure was deposited in the CSD as a private communication, so while it is possible there is an error (*e.g.* the wrong atom type) more information is not readily available and the structure is included in the plot in Fig. 5 ▸ for completeness.

## 4. Synthesis and crystallization

For the synthesis of AgPy_4_PF_6_ (**I**), silver nitrate (1.5 g, 8.83 mmol, 1 eq.) and potassium hexa­fluoro­phosphate (1.63 g, 8.83 mmol, 1 eq.) were dissolved in deionized water (15 ml). Pyridine (2.0 ml, 24.7 mmol, 2.8 eq.) was added dropwise and the solution was stirred for 1 h. The precipitate was collected by suction filtration, washed with copious amounts of deionized water and dried for two days in a desiccator. Crystals were grown by vapour diffusion using DCM as the solvent and petroleum ether as the anti-solvent. Similar crystals (with a statistically indistinguishable unit cell) were also found using MeOH as the anti-solvent.

AgPy_2_SbF_6_ (**II**) was synthesized directly from AgSbF_6_ (0.8 g, 2.33 mmol, 1 eq.) which was dissolved in deionized water (10 ml). Pyridine (0.53 ml, 6.52 mmol, 2.8 eq.) was added dropwise and the solution was stirred for 1 h. The precipitate was collected by suction filtration, washed with copious amounts of deionized water and dried for two days in a desiccator. Crystals were grown by solvent evaporation of DCM. Similar crystals (with a statistically indistinguishable unit cell) were also grown by vapour diffusion using DCM as the solvent and EtOH as the anti-solvent.

## 5. Refinement

Crystal data, data collection and structure refinement details are summarized in Table 5 ▸. Both **I** and **II** crystallized from a mixed phase mixture with the AgPy_2_*X* analogue. Suitable crystals were isolated and mounted on a MiTeGen loop using perfluoro­polyether oil and placed in the N_2_ stream of an Oxford CryoSystems CryoStream unit (Cosier & Glazer, 1986 ▸) at 300 K. Diffraction data were measured using a (Rigaku) Oxford Diffraction SuperNova A diffractometer (*K*α radiation, λ = 1.54184 Å). Raw frame images were processed using *CrysAlis PRO* (Rigaku OD, 2022 ▸). In both cases, the reflections were indexed using the same orientation matrix at both temperatures to enable comparison.

Examination of the symmetry equivalents and systematic absences suggested possible space groups of *I*4, *I*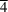 and *I*4/*m;* however, structure solution with charge flipping using *SUPERFLIP* (Palatinus & Chapuis, 2007 ▸) suggested the space group was *I*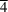. Once the symmetry and unit-cell contents had been confirmed, the crystal faces were indexed and an absorption correction applied.

The initial solution of the structure of **I** from the data collected at 300 K located all non-hydrogen atoms. Subsequent full-matrix least-squares refinement was carried out using the *CRYSTALS* program suite (Betteridge *et al.*, 2003 ▸). Coordinates and anisotropic displacement parameters of all non-hydrogen atoms were refined. The hydrogen atoms were all visible in the difference map, but were repositioned geometrically (Cooper *et al.*, 2010 ▸). Initially they were refined with soft restraints on the bond lengths and angles to regularize their geometry (C—H distance = 0.93 Å), and *U*_iso_(H) (1.2 times *U*_eq_ of the parent atom), after which the positions were refined with riding constraints.

The structure of **I** from the 300 K data was then refined against the data collected at 100 K: initially the non-hydrogen atoms were refined, then the hydrogen atoms with restraints before including them in the model with riding constraints. Similarly and to give a comparable set of structures, the structure of **I** at 300 K was refined against the data collected on **II** at 300 K. The model was modified to replace phospho­rus with anti­mony and it was refined as above. Finally, the model for **II** at 300 K was refined against the data collected at 100 K (also as above). It is of note that the *R*-indices for **II** refined against the 300 K data are higher than those for the structure refined against the 100 K data. This is thought to be due to the fact that the data are weaker at the higher temperature and therefore noisier, something that is reflected in the inter­nal agreement factors (5.3% and 300 K and 2.8% at 100 K).

Towards the end of each refinement, a modified Sheldrick weighting scheme was applied as per the details below (Watkin, 1994 ▸). In each case, the Flack *x* parameter (Flack, 1983 ▸) was included in the refinement and the absolute structure determined by analysis of the Bijvoet pairs (Thompson *et al.*, 2009 ▸; Parsons *et al.*, 2013 ▸). The void and dihedral angle calculations were carried out using *PLATON* (van der Sluis & Spek 1990 ▸; Spek, 2003 ▸, Spek 2009 ▸; Spek, 2015 ▸) and displacement ellipsoid plots were drawn with *CAMERON* (Watkin *et al.*, 1996 ▸).

## Supplementary Material

Crystal structure: contains datablock(s) I-300K, I-100K, II-300K, II-100K, global. DOI: 10.1107/S2056989024006972/oo2005sup1.cif

Structure factors: contains datablock(s) I-300K. DOI: 10.1107/S2056989024006972/oo2005I-300Ksup2.hkl

Structure factors: contains datablock(s) I-100K. DOI: 10.1107/S2056989024006972/oo2005I-100Ksup3.hkl

Structure factors: contains datablock(s) II-300K. DOI: 10.1107/S2056989024006972/oo2005II-300Ksup4.hkl

Structure factors: contains datablock(s) II-100K. DOI: 10.1107/S2056989024006972/oo2005II-100Ksup5.hkl

CCDC references: 2371460, 2371461, 2371462, 2371463

Additional supporting information:  crystallographic information; 3D view; checkCIF report

## Figures and Tables

**Figure 2:**
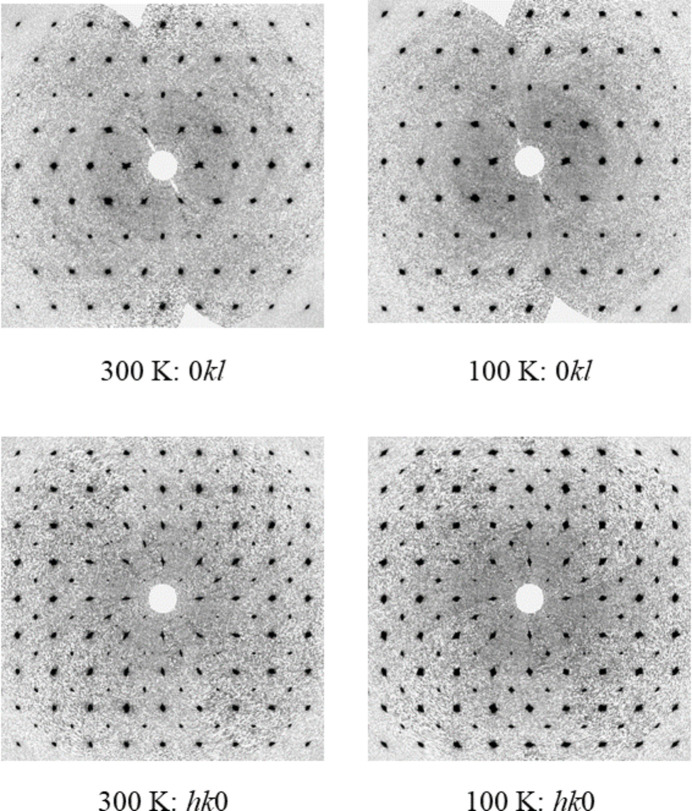
Selected reciprocal lattice sections reconstructed to 1.5 Å for the zero order layers for **II** at 300 K and 100 K.

**Figure 4:**
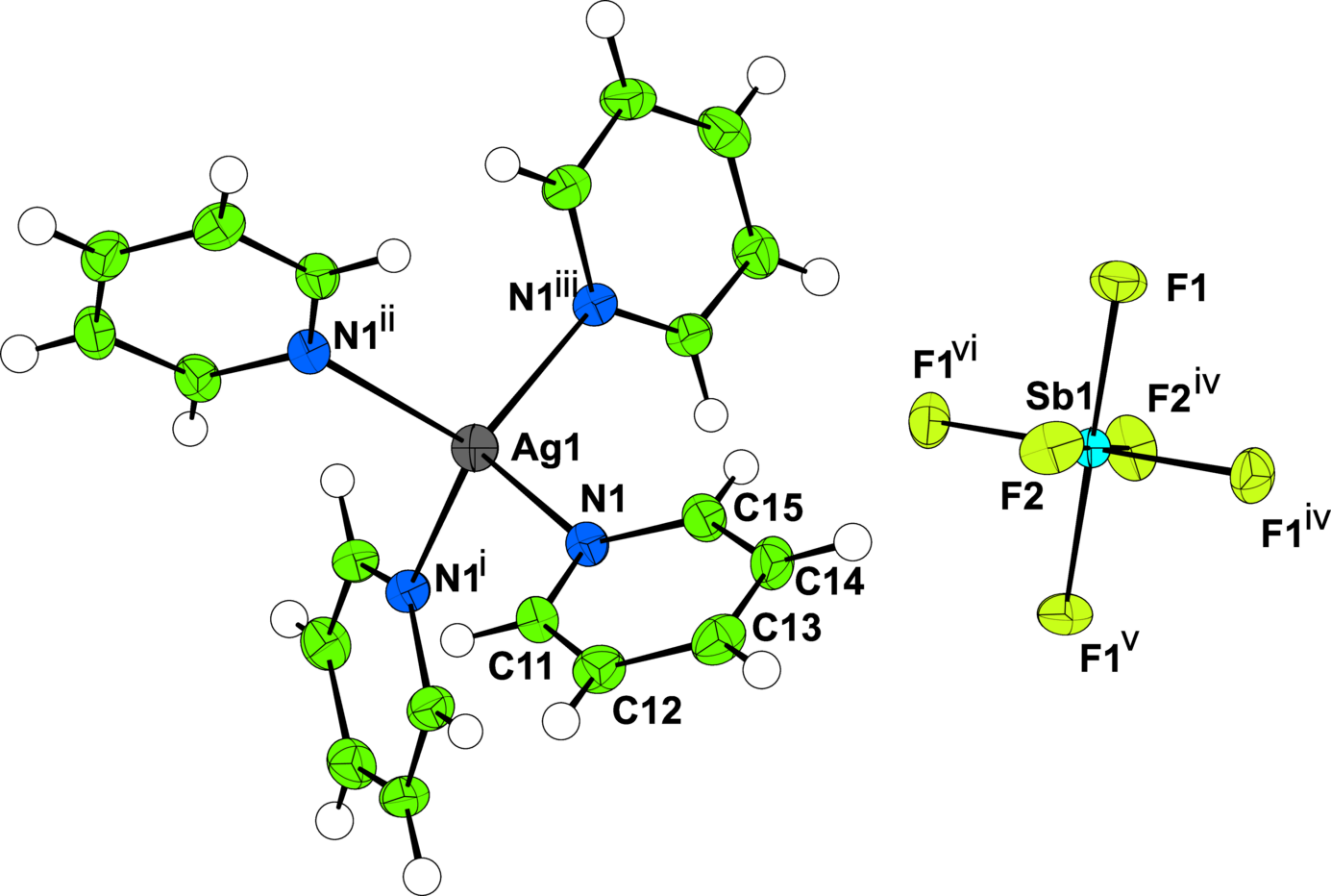
The mol­ecular structure of **II** at 100 K. Displacement ellipsoids are drawn at the 50% probability level. Symmetry codes: (i) *y*, 1 − *x*, 1 − *z*; (ii) 1 − *x*, 1 − *y*, *z*; (iii) 1 − *y*, *x*, 1 − *z*; (iv) *y* − 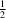, 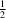 − *x*, 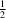 − *z*; (v) −*x*, 1 − *y*, *z*; (vi) 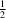 − *y*, 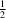 + *x*, 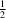 − *z*.

**Table 1:** Selected geometric parameters (Å, °) for **I** at 300 K[Chem scheme1]

Ag1—N1^i^	2.322 (3)	C13—C14	1.356 (6)
Ag1—N1^ii^	2.322 (3)	C14—C15	1.362 (6)
Ag1—N1^iii^	2.322 (3)	P1—F1^iv^	1.572 (2)
Ag1—N1	2.322 (3)	P1—F1^v^	1.572 (2)
N1—C11	1.318 (5)	P1—F1^vi^	1.572 (2)
N1—C15	1.331 (4)	P1—F2^iv^	1.556 (4)
C11—C12	1.354 (6)	P1—F1	1.572 (2)
C12—C13	1.363 (6)	P1—F2	1.556 (4)
			
N1^i^—Ag1—N1^ii^	106.90 (8)	F1^iv^—P1—F1^vi^	179.7 (2)
N1^i^—Ag1—N1^iii^	114.75 (16)	F1^v^—P1—F1^vi^	90.00
N1^ii^—Ag1—N1^iii^	106.90 (8)	F1^iv^—P1—F2^iv^	90.16 (12)
N1^i^—Ag1—N1	106.90 (8)	F1^v^—P1—F2^iv^	89.84 (12)
N1^ii^—Ag1—N1	114.75 (16)	F1^vi^—P1—F2^iv^	90.16 (12)
N1^iii^—Ag1—N1	106.90 (8)	F1^iv^—P1—F1	90.00
Ag1—N1—C11	121.2 (2)	F1^v^—P1—F1	179.7 (2)
Ag1—N1—C15	120.9 (3)	F1^vi^—P1—F1	90.00
C11—N1—C15	117.5 (3)	F2^iv^—P1—F1	89.84 (12)
N1—C11—C12	122.5 (4)	F1^iv^—P1—F2	89.84 (12)
C11—C12—C13	119.8 (4)	F1^v^—P1—F2	90.16 (12)
C12—C13—C14	118.3 (4)	F1^vi^—P1—F2	89.84 (12)
C13—C14—C15	118.9 (4)	F2^iv^—P1—F2	179.99
C14—C15—N1	122.9 (4)	F1—P1—F2	90.16 (12)
F1^iv^—P1—F1^v^	90.00		

Symmetry codes: (i) 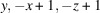; (ii) 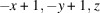; (iii) 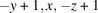; (iv) 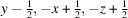; (v) 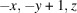; (vi) 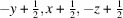.

**Table 2:** Selected geometric parameters (Å, °) for **I** at 100 K[Chem scheme1]

Ag1—N1^i^	2.310 (2)	C13—C14	1.381 (5)
Ag1—N1^ii^	2.310 (2)	C14—C15	1.392 (4)
Ag1—N1^iii^	2.310 (2)	P1—F1^iv^	1.6047 (15)
Ag1—N1	2.310 (2)	P1—F1^v^	1.6047 (15)
N1—C11	1.346 (3)	P1—F1^vi^	1.6047 (15)
N1—C15	1.343 (3)	P1—F2^iv^	1.584 (4)
C11—C12	1.381 (4)	P1—F1	1.6047 (15)
C12—C13	1.389 (4)	P1—F2	1.584 (4)
			
N1^i^—Ag1—N1^ii^	105.36 (5)	F1^iv^—P1—F1^vi^	179.88 (12)
N1^i^—Ag1—N1^iii^	105.36 (5)	F1^v^—P1—F1^vi^	90.00
N1^ii^—Ag1—N1^iii^	118.04 (10)	F1^iv^—P1—F2^iv^	90.06 (6)
N1^i^—Ag1—N1	118.04 (10)	F1^v^—P1—F2^iv^	89.94 (6)
N1^ii^—Ag1—N1	105.36 (5)	F1^vi^—P1—F2^iv^	90.06 (6)
N1^iii^—Ag1—N1	105.36 (5)	F1^iv^—P1—F1	90.00
Ag1—N1—C11	121.60 (16)	F1^v^—P1—F1	179.88 (12)
Ag1—N1—C15	120.02 (17)	F1^vi^—P1—F1	90.00
C11—N1—C15	117.5 (2)	F2^iv^—P1—F1	89.94 (6)
N1—C11—C12	122.9 (2)	F1^iv^—P1—F2	89.94 (6)
C11—C12—C13	119.2 (3)	F1^v^—P1—F2	90.06 (6)
C12—C13—C14	118.5 (3)	F1^vi^—P1—F2	89.94 (6)
C13—C14—C15	119.0 (2)	F2^iv^—P1—F2	179.99
C14—C15—N1	122.9 (2)	F1—P1—F2	90.06 (6)
F1^iv^—P1—F1^v^	90.00		

Symmetry codes: (i) 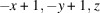; (ii) 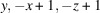; (iii) 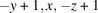; (iv) 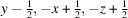; (v) 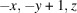; (vi) 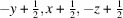.

**Table 3:** Selected geometric parameters (Å, °) for **II** at 300 K[Chem scheme1]

Ag1—N1^i^	2.324 (7)	C13—C14	1.370 (18)
Ag1—N1^ii^	2.324 (7)	C14—C15	1.366 (15)
Ag1—N1^iii^	2.324 (7)	Sb1—F2^iv^	1.872 (8)
Ag1—N1	2.324 (7)	Sb1—F1^v^	1.849 (5)
N1—C11	1.328 (12)	Sb1—F1^iv^	1.849 (5)
N1—C15	1.347 (10)	Sb1—F1^vi^	1.849 (5)
C11—C12	1.342 (15)	Sb1—F1	1.849 (5)
C12—C13	1.368 (17)	Sb1—F2	1.872 (8)
			
N1^i^—Ag1—N1^ii^	108.10 (18)	F2^iv^—Sb1—F1^iv^	92.0 (5)
N1^i^—Ag1—N1^iii^	108.10 (18)	F1^v^—Sb1—F1^iv^	176.1 (10)
N1^ii^—Ag1—N1^iii^	112.2 (4)	F2^iv^—Sb1—F1^vi^	88.0 (5)
N1^i^—Ag1—N1	112.2 (4)	F1^v^—Sb1—F1^vi^	90.07 (3)
N1^ii^—Ag1—N1	108.10 (18)	F1^iv^—Sb1—F1^vi^	90.07 (3)
N1^iii^—Ag1—N1	108.10 (18)	F2^iv^—Sb1—F1	88.0 (5)
Ag1—N1—C11	121.3 (5)	F1^v^—Sb1—F1	90.07 (3)
Ag1—N1—C15	121.0 (6)	F1^iv^—Sb1—F1	90.07 (3)
C11—N1—C15	117.6 (8)	F1^vi^—Sb1—F1	176.1 (10)
N1—C11—C12	123.2 (9)	F2^iv^—Sb1—F2	179.99
C11—C12—C13	120.1 (11)	F1^v^—Sb1—F2	88.0 (5)
C12—C13—C14	117.4 (10)	F1^iv^—Sb1—F2	88.0 (5)
C13—C14—C15	120.3 (9)	F1^vi^—Sb1—F2	92.0 (5)
C14—C15—N1	121.3 (9)	F1—Sb1—F2	92.0 (5)
F2^iv^—Sb1—F1^v^	92.0 (5)		

Symmetry codes: (i) 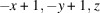; (ii) 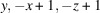; (iii) 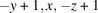; (iv) 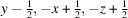; (v) 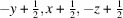; (vi) 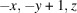.

**Table 4:** Selected geometric parameters (Å, °) for **II** at 100 K[Chem scheme1]

Ag1—N1^i^	2.305 (2)	C13—C14	1.384 (4)
Ag1—N1^ii^	2.305 (2)	C14—C15	1.385 (4)
Ag1—N1^iii^	2.305 (2)	Sb1—F2^iv^	1.8809 (17)
Ag1—N1	2.305 (2)	Sb1—F1^v^	1.8776 (13)
N1—C11	1.338 (3)	Sb1—F1^iv^	1.8776 (13)
N1—C15	1.348 (3)	Sb1—F1^vi^	1.8776 (13)
C11—C12	1.386 (4)	Sb1—F1	1.8776 (13)
C12—C13	1.383 (4)	Sb1—F2	1.8809 (17)
			
N1^i^—Ag1—N1^ii^	107.70 (5)	F2^iv^—Sb1—F1^iv^	90.62 (7)
N1^i^—Ag1—N1^iii^	107.70 (5)	F1^v^—Sb1—F1^iv^	178.75 (14)
N1^ii^—Ag1—N1^iii^	113.08 (11)	F2^iv^—Sb1—F1^vi^	89.38 (7)
N1^i^—Ag1—N1	113.08 (11)	F1^v^—Sb1—F1^vi^	90.01
N1^ii^—Ag1—N1	107.70 (5)	F1^iv^—Sb1—F1^vi^	90.01
N1^iii^—Ag1—N1	107.70 (5)	F2^iv^—Sb1—F1	89.38 (7)
Ag1—N1—C11	121.63 (17)	F1^v^—Sb1—F1	90.01
Ag1—N1—C15	120.93 (17)	F1^iv^—Sb1—F1	90.01
C11—N1—C15	117.1 (2)	F1^vi^—Sb1—F1	178.75 (14)
N1—C11—C12	123.3 (2)	F2^iv^—Sb1—F2	179.99
C11—C12—C13	118.9 (3)	F1^v^—Sb1—F2	89.38 (7)
C12—C13—C14	118.5 (2)	F1^iv^—Sb1—F2	89.38 (7)
C13—C14—C15	119.0 (2)	F1^vi^—Sb1—F2	90.62 (7)
C14—C15—N1	123.1 (3)	F1—Sb1—F2	90.62 (7)
F2^iv^—Sb1—F1^v^	90.62 (7)		

Symmetry codes: (i) 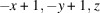; (ii) 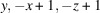; (iii) 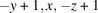; (iv) 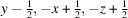; (v) 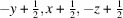; (vi) 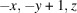.

**Table 5:** Experimental details

	**I** at 300 K	**I** at 100 K	**II** at 300 K	**II** at 100 K
Crystal data				
Chemical formula	[Ag(C_5_H_5_N)_4_](PF_6_)	[Ag(C_5_H_5_N)_4_](PF_6_)	[Ag(C_5_H_5_N)_4_](SbF_6_)	[Ag(C_5_H_5_N)_4_](SbF_6_)
*M*_r_	569.24	569.23	660.01	660.01
Crystal system, space group	Tetragonal, *I* 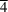	Tetragonal, *I* 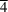	Tetragonal, *I* 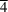	Tetragonal, *I* 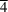
Temperature (K)	300	100	300	100
*a*, *c* (Å)	13.2831 (3), 6.6894 (7)	13.4408 (2), 6.1851 (1)	13.3825 (1), 6.8847 (1)	13.3461 (1), 6.5798 (1)
*V* (Å^3^)	1180.28 (13)	1117.37 (4)	1232.99 (3)	1171.98 (3)
*Z*	2	2	2	2
Radiation type	Cu *K*α	Cu *K*α	Cu *K*α	Cu *K*α
μ (mm^−1^)	8.06	8.52	15.60	16.42
Crystal size (mm)	0.23 × 0.06 × 0.05	0.23 × 0.06 × 0.05	0.25 × 0.20 × 0.05	0.25 × 0.20 × 0.05

Data collection				
Diffractometer	Oxford Diffraction SuperNova A	Oxford Diffraction SuperNova A	Oxford Diffraction SuperNova A	Oxford Diffraction SuperNova A
Absorption correction	Gaussian (*CrysAlis PRO*; Rigaku OD, 2022 ▸)	Gaussian (*CrysAlis PRO*; Rigaku OD, 2022 ▸)	Gaussian (*CrysAlis PRO*; Rigaku OD, 2022 ▸)	Gaussian (*CrysAlis PRO*; Rigaku OD, 2022 ▸)
*T*_min_, *T*_max_	0.499, 1.000	0.153, 0.627	0.012, 0.394	0.093, 0.623
No. of measured, independent and observed [*I* > 2.0σ(*I*)] reflections	12427, 1240, 1154	10819, 1162, 1153	5859, 1286, 1281	5473, 1219, 1215
*R*_int_	0.044	0.054	0.053	0.028
(sin θ/λ)_max_ (Å^−1^)	0.629	0.629	0.628	0.629

Refinement				
*R*[*F*^2^ > 2σ(*F*^2^)], *wR*(*F*^2^), *S*	0.028, 0.065, 0.86	0.021, 0.028, 1.01	0.048, 0.132, 1.01	0.017, 0.045, 1.02
No. of reflections	1240	1162	1286	1219
No. of parameters	73	73	73	73
H-atom treatment	H-atom parameters constrained	H-atom parameters constrained	H-atom parameters constrained	H-atom parameters constrained
Δρ_max_, Δρ_min_ (e Å^−3^)	0.15, −0.30	0.58, −0.75	0.27, −0.73	0.20, −0.30
Absolute structure	Parsons *et al.* (2013 ▸), 469 Friedel Pairs	Parsons *et al.* (2013 ▸), 456 Friedel Pairs	Parsons *et al.* (2013 ▸), 471 Friedel Pairs	Parsons *et al.* (2013 ▸), 469 Friedel Pairs
Absolute structure parameter	−0.020 (5)	−0.028 (4)	−0.022 (17)	−0.006 (5)

Computer programs: *CrysAlis PRO* (Rigaku OD, 2022 ▸), *SUPERFLIP* (Palatinus & Chapuis, 2007 ▸), *CRYSTALS* (Betteridge *et al.*, 2003 ▸) and *CAMERON* (Watkin *et al.*, 1996 ▸).

## supplementary crystallographic information

### Tetrapyridinesilver(I) hexafluorophosphate (I-300K) Crystal data

**Table:**

[Ag(C_5_H_5_N)_4_](PF_6_)	*D*_x_ = 1.602 Mg m^−^^3^
*M**_r_* = 569.24	Melting point: not measured K
Tetragonal, *I*4	Cu *K*α radiation, λ = 1.54184 Å
Hall symbol: I -4	Cell parameters from 3328 reflections
*a* = 13.2831 (3) Å	θ = 4.7–73.0°
*c* = 6.6894 (7) Å	µ = 8.06 mm^−^^1^
*V* = 1180.28 (13) Å^3^	*T* = 300 K
*Z* = 2	Needle, clear_pale_colourless
*F*(000) = 568	0.23 × 0.06 × 0.05 mm

### Tetrapyridinesilver(I) hexafluorophosphate (I-300K) Data collection

**Table:**

Oxford Diffraction SuperNova A diffractometer	1154 reflections with *I* > 2.0σ(*I*)
Focussing mirrors monochromator	*R*_int_ = 0.044
ω scans	θ_max_ = 76.0°, θ_min_ = 4.7°
Absorption correction: gaussian (CrysAlisPro; Rigaku OD, 2022)	*h* = −15→16
*T*_min_ = 0.499, *T*_max_ = 1.000	*k* = −16→16
12427 measured reflections	*l* = −8→8
1240 independent reflections	

### Tetrapyridinesilver(I) hexafluorophosphate (I-300K) Refinement

**Table:**

Refinement on *F*^2^	Hydrogen site location: difference Fourier map
Least-squares matrix: full	H-atom parameters constrained
*R*[*F*^2^ > 2σ(*F*^2^)] = 0.028	Method = Modified Sheldrick *w* = 1/[σ^2^(*F*^2^) + ( 0.03*P*)^2^ + 1.19*P*] , where *P* = (max(*F*_o_^2^,0) + 2*F*_c_^2^)/3
*wR*(*F*^2^) = 0.065	(Δ/σ)_max_ = 0.0001
*S* = 0.86	Δρ_max_ = 0.15 e Å^−^^3^
1240 reflections	Δρ_min_ = −0.30 e Å^−^^3^
73 parameters	Absolute structure: Parsons *et al.* (2013), 469 Friedel Pairs
0 restraints	Absolute structure parameter: −0.020 (5)
Primary atom site location: other	

### Tetrapyridinesilver(I) hexafluorophosphate (I-300K) Special details

**Table:**

Refinement. Reflections are selected by the following conditions :- Minimum value of SQRTW is 0.00 Minimum value of RATIO is -3.00

### Tetrapyridinesilver(I) hexafluorophosphate (I-300K) Fractional atomic coordinates and isotropic or equivalent isotropic displacement parameters (Å^2^)

**Table:**

	*x*	*y*	*z*	*U*_iso_*/*U*_eq_	
Ag1	0.5000	0.5000	0.5000	0.0932	
N1	0.3770 (2)	0.5809 (2)	0.6871 (5)	0.0789	
C11	0.4011 (3)	0.6450 (3)	0.8296 (7)	0.0917	
C12	0.3323 (4)	0.6830 (3)	0.9585 (8)	0.1104	
C13	0.2339 (4)	0.6548 (4)	0.9433 (8)	0.1110	
C14	0.2075 (3)	0.5911 (3)	0.7940 (8)	0.1002	
C15	0.2804 (3)	0.5557 (3)	0.6692 (7)	0.0875	
P1	0.0000	0.5000	0.2500	0.0703	
F1	−0.0213 (2)	0.38358 (17)	0.2493 (5)	0.1276	
F2	0.0000	0.5000	0.4827 (7)	0.1263	
H111	0.4693	0.6662	0.8390	0.1101*	
H121	0.3514	0.7252	1.0625	0.1420*	
H131	0.1855	0.6797	1.0331	0.1380*	
H141	0.1398	0.5717	0.7759	0.1199*	
H151	0.2620	0.5117	0.5652	0.1029*	

### Tetrapyridinesilver(I) hexafluorophosphate (I-300K) Atomic displacement parameters (Å^2^)

**Table:**

	*U*^11^	*U*^22^	*U*^33^	*U*^12^	*U*^13^	*U*^23^
Ag1	0.0809 (2)	0.0809 (2)	0.1176 (4)	0.0000	0.0000	0.0000
N1	0.0643 (15)	0.0762 (17)	0.096 (2)	−0.0041 (12)	0.0023 (14)	−0.0071 (15)
C11	0.074 (2)	0.090 (2)	0.110 (3)	−0.0056 (18)	−0.008 (2)	−0.020 (2)
C12	0.117 (3)	0.108 (3)	0.106 (4)	0.010 (2)	−0.003 (3)	−0.026 (3)
C13	0.107 (3)	0.103 (3)	0.122 (4)	0.023 (2)	0.028 (3)	0.005 (3)
C14	0.068 (2)	0.097 (3)	0.135 (4)	−0.0037 (19)	0.012 (2)	0.011 (3)
C15	0.069 (2)	0.082 (2)	0.111 (3)	−0.0105 (17)	−0.0020 (19)	−0.009 (2)
P1	0.0686 (5)	0.0686 (5)	0.0738 (10)	0.0000	0.0000	0.0000
F1	0.152 (2)	0.0723 (14)	0.158 (2)	−0.0152 (15)	−0.001 (2)	−0.0115 (15)
F2	0.176 (3)	0.132 (3)	0.0702 (18)	0.007 (2)	0.0000	0.0000

### Tetrapyridinesilver(I) hexafluorophosphate (I-300K) Geometric parameters (Å, º)

**Table:**

Ag1—N1^i^	2.322 (3)	C13—H131	0.940
Ag1—N1^ii^	2.322 (3)	C14—C15	1.362 (6)
Ag1—N1^iii^	2.322 (3)	C14—H141	0.944
Ag1—N1	2.322 (3)	C15—H151	0.940
N1—C11	1.318 (5)	P1—F1^iv^	1.572 (2)
N1—C15	1.331 (4)	P1—F1^v^	1.572 (2)
C11—C12	1.354 (6)	P1—F1^vi^	1.572 (2)
C11—H111	0.951	P1—F2^iv^	1.556 (4)
C12—C13	1.363 (6)	P1—F1	1.572 (2)
C12—H121	0.929	P1—F2	1.556 (4)
C13—C14	1.356 (6)		
			
N1^i^—Ag1—N1^ii^	106.90 (8)	C15—C14—H141	120.3
N1^i^—Ag1—N1^iii^	114.75 (16)	C14—C15—N1	122.9 (4)
N1^ii^—Ag1—N1^iii^	106.90 (8)	C14—C15—H151	118.9
N1^i^—Ag1—N1	106.90 (8)	N1—C15—H151	118.2
N1^ii^—Ag1—N1	114.75 (16)	F1^iv^—P1—F1^v^	90.00
N1^iii^—Ag1—N1	106.90 (8)	F1^iv^—P1—F1^vi^	179.7 (2)
Ag1—N1—C11	121.2 (2)	F1^v^—P1—F1^vi^	90.00
Ag1—N1—C15	120.9 (3)	F1^iv^—P1—F2^iv^	90.16 (12)
C11—N1—C15	117.5 (3)	F1^v^—P1—F2^iv^	89.84 (12)
N1—C11—C12	122.5 (4)	F1^vi^—P1—F2^iv^	90.16 (12)
N1—C11—H111	118.1	F1^iv^—P1—F1	90.00
C12—C11—H111	119.4	F1^v^—P1—F1	179.7 (2)
C11—C12—C13	119.8 (4)	F1^vi^—P1—F1	90.00
C11—C12—H121	121.2	F2^iv^—P1—F1	89.84 (12)
C13—C12—H121	118.9	F1^iv^—P1—F2	89.84 (12)
C12—C13—C14	118.3 (4)	F1^v^—P1—F2	90.16 (12)
C12—C13—H131	120.8	F1^vi^—P1—F2	89.84 (12)
C14—C13—H131	120.9	F2^iv^—P1—F2	179.99
C13—C14—C15	118.9 (4)	F1—P1—F2	90.16 (12)
C13—C14—H141	120.8		

Symmetry codes: (i) *y*, −*x*+1, −*z*+1; (ii) −*x*+1, −*y*+1, *z*; (iii) −*y*+1, *x*, −*z*+1; (iv) *y*−1/2, −*x*+1/2, −*z*+1/2; (v) −*x*, −*y*+1, *z*; (vi) −*y*+1/2, *x*+1/2, −*z*+1/2.

### Tetrapyridinesilver(I) hexafluorophosphate (I-100K) Crystal data

**Table:**

[Ag(C_5_H_5_N)_4_](PF_6_)	*D*_x_ = 1.692 Mg m^−^^3^
*M**_r_* = 569.23	Melting point: not measured K
Tetragonal, *I*4	Cu *K*α radiation, λ = 1.54184 Å
Hall symbol: I -4	Cell parameters from 8381 reflections
*a* = 13.4408 (2) Å	θ = 4.6–75.8°
*c* = 6.1851 (1) Å	µ = 8.52 mm^−^^1^
*V* = 1117.37 (4) Å^3^	*T* = 100 K
*Z* = 2	Needle, clear_pale_colourless
*F*(000) = 567.998	0.23 × 0.06 × 0.05 mm

### Tetrapyridinesilver(I) hexafluorophosphate (I-100K) Data collection

**Table:**

Oxford Diffraction SuperNova A diffractometer	1153 reflections with *I* > 2.0σ(*I*)
Focussing mirrors monochromator	*R*_int_ = 0.054
ω scans	θ_max_ = 75.9°, θ_min_ = 4.7°
Absorption correction: gaussian (CrysAlisPro; Rigaku OD, 2022)	*h* = −15→16
*T*_min_ = 0.153, *T*_max_ = 0.627	*k* = −16→16
10819 measured reflections	*l* = −7→7
1162 independent reflections	

### Tetrapyridinesilver(I) hexafluorophosphate (I-100K) Refinement

**Table:**

Refinement on *F*	Hydrogen site location: difference Fourier map
Least-squares matrix: full	H-atom parameters constrained
*R*[*F*^2^ > 2σ(*F*^2^)] = 0.021	Method = Modified Sheldrick *w* = 1/[σ^2^(*F*) + ( 0.02*P*)^2^ + 0.0*P*] , where *P* = (max(*F*_o_,0) + 2*F*_c_)/3
*wR*(*F*^2^) = 0.028	(Δ/σ)_max_ = 0.0001
*S* = 1.01	Δρ_max_ = 0.58 e Å^−^^3^
1162 reflections	Δρ_min_ = −0.75 e Å^−^^3^
73 parameters	Absolute structure: Parsons *et al.* (2013), 456 Friedel Pairs
0 restraints	Absolute structure parameter: −0.028 (4)
Primary atom site location: other	

### Tetrapyridinesilver(I) hexafluorophosphate (I-100K) Special details

**Table:**

Refinement. Reflections are selected by the following conditions :- Minimum value of SQRTW is 0.00 Minimum value of RATIO is -3.00

### Tetrapyridinesilver(I) hexafluorophosphate (I-100K) Fractional atomic coordinates and isotropic or equivalent isotropic displacement parameters (Å^2^)

**Table:**

	*x*	*y*	*z*	*U*_iso_*/*U*_eq_	
Ag1	0.5000	0.5000	0.5000	0.0318	
N1	0.37821 (15)	0.58293 (16)	0.6922 (3)	0.0292	
C11	0.40105 (19)	0.64517 (19)	0.8554 (4)	0.0310	
C12	0.33091 (17)	0.68141 (16)	0.9983 (8)	0.0375	
C13	0.23219 (19)	0.65286 (19)	0.9749 (7)	0.0393	
C14	0.2074 (2)	0.5906 (2)	0.8057 (5)	0.0368	
C15	0.28217 (19)	0.55778 (19)	0.6672 (4)	0.0328	
P1	0.0000	0.5000	0.2500	0.0269	
F1	−0.02173 (13)	0.38260 (11)	0.2497 (3)	0.0383	
F2	0.0000	0.5000	0.5060 (6)	0.0413	
H111	0.4677	0.6654	0.8686	0.0365*	
H121	0.3499	0.7242	1.1087	0.0446*	
H131	0.1831	0.6757	1.0704	0.0470*	
H141	0.1426	0.5711	0.7848	0.0442*	
H151	0.2655	0.5165	0.5510	0.0385*	

### Tetrapyridinesilver(I) hexafluorophosphate (I-100K) Atomic displacement parameters (Å^2^)

**Table:**

	*U*^11^	*U*^22^	*U*^33^	*U*^12^	*U*^13^	*U*^23^
Ag1	0.03228 (12)	0.03228 (12)	0.03097 (15)	0.0000	0.0000	0.0000
N1	0.0253 (9)	0.0323 (10)	0.0301 (10)	−0.0012 (7)	−0.0007 (7)	−0.0011 (8)
C11	0.0294 (11)	0.0327 (12)	0.0307 (11)	−0.0001 (9)	−0.0018 (9)	−0.0023 (9)
C12	0.0424 (11)	0.0341 (9)	0.0359 (10)	0.0042 (8)	0.0015 (19)	−0.0028 (19)
C13	0.0393 (11)	0.0373 (11)	0.0414 (19)	0.0091 (9)	0.0107 (13)	0.0070 (13)
C14	0.0279 (11)	0.0363 (12)	0.0463 (15)	0.0003 (9)	0.0033 (10)	0.0079 (10)
C15	0.0304 (11)	0.0317 (11)	0.0364 (11)	−0.0029 (9)	−0.0020 (10)	0.0013 (10)
P1	0.0291 (3)	0.0291 (3)	0.0223 (6)	0.0000	0.0000	0.0000
F1	0.0416 (8)	0.0310 (7)	0.0422 (8)	−0.0018 (6)	−0.0014 (7)	−0.0054 (6)
F2	0.0609 (11)	0.0387 (9)	0.0243 (9)	−0.0006 (8)	−0.0000	−0.0000

### Tetrapyridinesilver(I) hexafluorophosphate (I-100K) Geometric parameters (Å, º)

**Table:**

Ag1—N1^i^	2.310 (2)	C13—H131	0.938
Ag1—N1^ii^	2.310 (2)	C14—C15	1.392 (4)
Ag1—N1^iii^	2.310 (2)	C14—H141	0.919
Ag1—N1	2.310 (2)	C15—H151	0.935
N1—C11	1.346 (3)	P1—F1^iv^	1.6047 (15)
N1—C15	1.343 (3)	P1—F1^v^	1.6047 (15)
C11—C12	1.381 (4)	P1—F1^vi^	1.6047 (15)
C11—H111	0.939	P1—F2^iv^	1.584 (4)
C12—C13	1.389 (4)	P1—F1	1.6047 (15)
C12—H121	0.929	P1—F2	1.584 (4)
C13—C14	1.381 (5)		
			
N1^i^—Ag1—N1^ii^	105.36 (5)	C15—C14—H141	120.5
N1^i^—Ag1—N1^iii^	105.36 (5)	C14—C15—N1	122.9 (2)
N1^ii^—Ag1—N1^iii^	118.04 (10)	C14—C15—H151	119.2
N1^i^—Ag1—N1	118.04 (10)	N1—C15—H151	117.9
N1^ii^—Ag1—N1	105.36 (5)	F1^iv^—P1—F1^v^	90.00
N1^iii^—Ag1—N1	105.36 (5)	F1^iv^—P1—F1^vi^	179.88 (12)
Ag1—N1—C11	121.60 (16)	F1^v^—P1—F1^vi^	90.00
Ag1—N1—C15	120.02 (17)	F1^iv^—P1—F2^iv^	90.06 (6)
C11—N1—C15	117.5 (2)	F1^v^—P1—F2^iv^	89.94 (6)
N1—C11—C12	122.9 (2)	F1^vi^—P1—F2^iv^	90.06 (6)
N1—C11—H111	117.5	F1^iv^—P1—F1	90.00
C12—C11—H111	119.6	F1^v^—P1—F1	179.88 (12)
C11—C12—C13	119.2 (3)	F1^vi^—P1—F1	90.00
C11—C12—H121	120.1	F2^iv^—P1—F1	89.94 (6)
C13—C12—H121	120.7	F1^iv^—P1—F2	89.94 (6)
C12—C13—C14	118.5 (3)	F1^v^—P1—F2	90.06 (6)
C12—C13—H131	121.1	F1^vi^—P1—F2	89.94 (6)
C14—C13—H131	120.4	F2^iv^—P1—F2	179.99
C13—C14—C15	119.0 (2)	F1—P1—F2	90.06 (6)
C13—C14—H141	120.5		

Symmetry codes: (i) −*x*+1, −*y*+1, *z*; (ii) *y*, −*x*+1, −*z*+1; (iii) −*y*+1, *x*, −*z*+1; (iv) *y*−1/2, −*x*+1/2, −*z*+1/2; (v) −*x*, −*y*+1, *z*; (vi) −*y*+1/2, *x*+1/2, −*z*+1/2.

### Tetrapyridinesilver(I) hexafluoroantimonate (II-300K) Crystal data

**Table:**

[Ag(C_5_H_5_N)_4_](SbF_6_)	*D*_x_ = 1.778 Mg m^−^^3^
*M**_r_* = 660.01	Melting point: not measured K
Tetragonal, *I*4	Cu *K*α radiation, λ = 1.54184 Å
Hall symbol: I -4	Cell parameters from 4713 reflections
*a* = 13.3825 (1) Å	θ = 4.7–75.2°
*c* = 6.8847 (1) Å	µ = 15.60 mm^−^^1^
*V* = 1232.99 (3) Å^3^	*T* = 300 K
*Z* = 2	Block, clear_pale_colourless
*F*(000) = 640	0.25 × 0.20 × 0.05 mm

### Tetrapyridinesilver(I) hexafluoroantimonate (II-300K) Data collection

**Table:**

Oxford Diffraction SuperNova A diffractometer	1281 reflections with *I* > 2.0σ(*I*)
Focussing mirrors monochromator	*R*_int_ = 0.053
ω scans	θ_max_ = 75.4°, θ_min_ = 4.7°
Absorption correction: gaussian (CrysAlisPro; Rigaku OD, 2022)	*h* = −16→16
*T*_min_ = 0.012, *T*_max_ = 0.394	*k* = −16→16
5859 measured reflections	*l* = −6→8
1286 independent reflections	

### Tetrapyridinesilver(I) hexafluoroantimonate (II-300K) Refinement

**Table:**

Refinement on *F*^2^	Hydrogen site location: difference Fourier map
Least-squares matrix: full	H-atom parameters constrained
*R*[*F*^2^ > 2σ(*F*^2^)] = 0.048	Method = Modified Sheldrick *w* = 1/[σ^2^(*F*^2^) + ( 0.11*P*)^2^ + 0.34*P*] , where *P* = (max(*F*_o_^2^,0) + 2*F*_c_^2^)/3
*wR*(*F*^2^) = 0.132	(Δ/σ)_max_ = 0.001
*S* = 1.00	Δρ_max_ = 0.27 e Å^−^^3^
1286 reflections	Δρ_min_ = −0.73 e Å^−^^3^
73 parameters	Absolute structure: Parsons *et al.* (2013), 471 Friedel Pairs
0 restraints	Absolute structure parameter: −0.022 (17)
Primary atom site location: other	

### Tetrapyridinesilver(I) hexafluoroantimonate (II-300K) Special details

**Table:**

Refinement. Reflections are selected by the following conditions :- Minimum value of SQRTW is 0.00 Minimum value of RATIO is -3.00

### Tetrapyridinesilver(I) hexafluoroantimonate (II-300K) Fractional atomic coordinates and isotropic or equivalent isotropic displacement parameters (Å^2^)

**Table:**

	*x*	*y*	*z*	*U*_iso_*/*U*_eq_	
Ag1	0.5000	0.5000	0.5000	0.1126	
N1	0.3792 (5)	0.5787 (5)	0.6882 (11)	0.0930	
C11	0.4043 (6)	0.6419 (8)	0.8286 (16)	0.1051	
C12	0.3377 (9)	0.6818 (10)	0.9521 (19)	0.1247	
C13	0.2384 (9)	0.6589 (9)	0.9356 (19)	0.1200	
C14	0.2110 (7)	0.5962 (8)	0.788 (2)	0.1162	
C15	0.2815 (6)	0.5573 (7)	0.6658 (17)	0.1041	
Sb1	0.0000	0.5000	0.2500	0.0747	
F1	−0.0202 (7)	0.3634 (4)	0.241 (2)	0.1602	
F2	0.0000	0.5000	0.5220 (11)	0.1412	
H111	0.4712	0.6581	0.8442	0.1228*	
H121	0.3593	0.7260	1.0471	0.1460*	
H131	0.1927	0.6855	1.0236	0.1440*	
H141	0.1443	0.5789	0.7713	0.1399*	
H151	0.2615	0.5158	0.5654	0.1219*	

### Tetrapyridinesilver(I) hexafluoroantimonate (II-300K) Atomic displacement parameters (Å^2^)

**Table:**

	*U*^11^	*U*^22^	*U*^33^	*U*^12^	*U*^13^	*U*^23^
Ag1	0.0990 (5)	0.0990 (5)	0.1398 (11)	0.0000	0.0000	0.0000
N1	0.078 (3)	0.091 (3)	0.110 (5)	−0.006 (3)	−0.003 (2)	−0.003 (3)
C11	0.086 (4)	0.108 (5)	0.122 (6)	−0.008 (4)	−0.009 (4)	−0.014 (4)
C12	0.128 (7)	0.133 (8)	0.114 (7)	0.001 (6)	−0.006 (6)	−0.024 (6)
C13	0.118 (6)	0.116 (6)	0.125 (7)	0.011 (5)	0.024 (6)	0.002 (5)
C14	0.084 (4)	0.128 (6)	0.137 (12)	−0.005 (4)	0.010 (5)	0.018 (6)
C15	0.086 (4)	0.103 (5)	0.123 (6)	−0.013 (4)	−0.010 (4)	−0.002 (4)
Sb1	0.0768 (3)	0.0768 (3)	0.0707 (3)	0.0000	0.0000	0.0000
F1	0.225 (8)	0.083 (2)	0.173 (6)	−0.021 (3)	0.012 (9)	−0.013 (5)
F2	0.188 (12)	0.148 (10)	0.088 (3)	0.025 (10)	0.0000	0.0000

### Tetrapyridinesilver(I) hexafluoroantimonate (II-300K) Geometric parameters (Å, º)

**Table:**

Ag1—N1^i^	2.324 (7)	C13—H131	0.932
Ag1—N1^ii^	2.324 (7)	C14—C15	1.366 (15)
Ag1—N1^iii^	2.324 (7)	C14—H141	0.929
Ag1—N1	2.324 (7)	C15—H151	0.926
N1—C11	1.328 (12)	Sb1—F2^iv^	1.872 (8)
N1—C15	1.347 (10)	Sb1—F1^v^	1.849 (5)
C11—C12	1.342 (15)	Sb1—F1^iv^	1.849 (5)
C11—H111	0.927	Sb1—F1^vi^	1.849 (5)
C12—C13	1.368 (17)	Sb1—F1	1.849 (5)
C12—H121	0.928	Sb1—F2	1.872 (8)
C13—C14	1.370 (18)		
			
N1^i^—Ag1—N1^ii^	108.10 (18)	C15—C14—H141	119.6
N1^i^—Ag1—N1^iii^	108.10 (18)	C14—C15—N1	121.3 (9)
N1^ii^—Ag1—N1^iii^	112.2 (4)	C14—C15—H151	119.1
N1^i^—Ag1—N1	112.2 (4)	N1—C15—H151	119.6
N1^ii^—Ag1—N1	108.10 (18)	F2^iv^—Sb1—F1^v^	92.0 (5)
N1^iii^—Ag1—N1	108.10 (18)	F2^iv^—Sb1—F1^iv^	92.0 (5)
Ag1—N1—C11	121.3 (5)	F1^v^—Sb1—F1^iv^	176.1 (10)
Ag1—N1—C15	121.0 (6)	F2^iv^—Sb1—F1^vi^	88.0 (5)
C11—N1—C15	117.6 (8)	F1^v^—Sb1—F1^vi^	90.07 (3)
N1—C11—C12	123.2 (9)	F1^iv^—Sb1—F1^vi^	90.07 (3)
N1—C11—H111	118.5	F2^iv^—Sb1—F1	88.0 (5)
C12—C11—H111	118.3	F1^v^—Sb1—F1	90.07 (3)
C11—C12—C13	120.1 (11)	F1^iv^—Sb1—F1	90.07 (3)
C11—C12—H121	119.6	F1^vi^—Sb1—F1	176.1 (10)
C13—C12—H121	120.3	F2^iv^—Sb1—F2	179.99
C12—C13—C14	117.4 (10)	F1^v^—Sb1—F2	88.0 (5)
C12—C13—H131	119.9	F1^iv^—Sb1—F2	88.0 (5)
C14—C13—H131	122.7	F1^vi^—Sb1—F2	92.0 (5)
C13—C14—C15	120.3 (9)	F1—Sb1—F2	92.0 (5)
C13—C14—H141	120.1		

Symmetry codes: (i) −*x*+1, −*y*+1, *z*; (ii) *y*, −*x*+1, −*z*+1; (iii) −*y*+1, *x*, −*z*+1; (iv) *y*−1/2, −*x*+1/2, −*z*+1/2; (v) −*y*+1/2, *x*+1/2, −*z*+1/2; (vi) −*x*, −*y*+1, *z*.

### Tetrapyridinesilver(I) hexafluoroantimonate (II-100K) Crystal data

**Table:**

[Ag(C_5_H_5_N)_4_](SbF_6_)	*D*_x_ = 1.870 Mg m^−^^3^
*M**_r_* = 660.01	Melting point: not measured K
Tetragonal, *I*4	Cu *K*α radiation, λ = 1.54184 Å
Hall symbol: I -4	Cell parameters from 4749 reflections
*a* = 13.3461 (1) Å	θ = 4.7–75.5°
*c* = 6.5798 (1) Å	µ = 16.42 mm^−^^1^
*V* = 1171.98 (3) Å^3^	*T* = 100 K
*Z* = 2	Block, clear_pale_colourless
*F*(000) = 640	0.25 × 0.20 × 0.05 mm

### Tetrapyridinesilver(I) hexafluoroantimonate (II-100K) Data collection

**Table:**

Oxford Diffraction SuperNova A diffractometer	1215 reflections with *I* > 2.0σ(*I*)
Focussing mirrors monochromator	*R*_int_ = 0.028
ω scans	θ_max_ = 75.9°, θ_min_ = 4.7°
Absorption correction: gaussian (CrysAlisPro; Rigaku OD, 2022)	*h* = −16→16
*T*_min_ = 0.093, *T*_max_ = 0.623	*k* = −16→16
5473 measured reflections	*l* = −6→8
1219 independent reflections	

### Tetrapyridinesilver(I) hexafluoroantimonate (II-100K) Refinement

**Table:**

Refinement on *F*^2^	Hydrogen site location: difference Fourier map
Least-squares matrix: full	H-atom parameters constrained
*R*[*F*^2^ > 2σ(*F*^2^)] = 0.017	Method = Modified Sheldrick *w* = 1/[σ^2^(*F*^2^) + ( 0.04*P*)^2^ + 0.43*P*] , where *P* = (max(*F*_o_^2^,0) + 2*F*_c_^2^)/3
*wR*(*F*^2^) = 0.045	(Δ/σ)_max_ = 0.0001
*S* = 1.02	Δρ_max_ = 0.20 e Å^−^^3^
1219 reflections	Δρ_min_ = −0.30 e Å^−^^3^
73 parameters	Absolute structure: Parsons *et al.* (2013), 469 Friedel Pairs
0 restraints	Absolute structure parameter: −0.006 (5)
Primary atom site location: other	

### Tetrapyridinesilver(I) hexafluoroantimonate (II-100K) Special details

**Table:**

Refinement. ? Reflections are selected by the following conditions :- Minimum value of SQRTW is 0.00 Minimum value of RATIO is -3.00

### Tetrapyridinesilver(I) hexafluoroantimonate (II-100K) Fractional atomic coordinates and isotropic or equivalent isotropic displacement parameters (Å^2^)

**Table:**

	*x*	*y*	*z*	*U*_iso_*/*U*_eq_	
Ag1	0.5000	0.5000	0.5000	0.0290	
N1	0.38043 (16)	0.58038 (16)	0.6931 (3)	0.0259	
C11	0.40561 (19)	0.64332 (19)	0.8429 (4)	0.0278	
C12	0.3366 (2)	0.6848 (2)	0.9760 (4)	0.0330	
C13	0.2365 (2)	0.6601 (2)	0.9552 (5)	0.0351	
C14	0.2089 (2)	0.5962 (2)	0.7993 (4)	0.0338	
C15	0.2824 (2)	0.5584 (2)	0.6721 (4)	0.0292	
Sb1	0.0000	0.5000	0.2500	0.0191	
F1	−0.02296 (12)	0.36121 (10)	0.2469 (3)	0.0356	
F2	0.0000	0.5000	0.5359 (3)	0.0370	
H111	0.4727	0.6606	0.8569	0.0342*	
H121	0.3572	0.7282	1.0784	0.0430*	
H131	0.1887	0.6854	1.0453	0.0441*	
H141	0.1420	0.5786	0.7805	0.0439*	
H151	0.2632	0.5160	0.5661	0.0370*	

### Tetrapyridinesilver(I) hexafluoroantimonate (II-100K) Atomic displacement parameters (Å^2^)

**Table:**

	*U*^11^	*U*^22^	*U*^33^	*U*^12^	*U*^13^	*U*^23^
Ag1	0.02868 (12)	0.02868 (12)	0.02968 (18)	0.0000	0.0000	0.0000
N1	0.0238 (10)	0.0259 (10)	0.0279 (10)	−0.0013 (8)	0.0004 (7)	0.0000 (7)
C11	0.0238 (11)	0.0280 (12)	0.0315 (12)	−0.0017 (9)	−0.0022 (9)	0.0000 (10)
C12	0.0371 (13)	0.0308 (12)	0.0311 (13)	0.0035 (10)	0.0002 (11)	−0.0049 (11)
C13	0.0343 (13)	0.0341 (13)	0.0368 (16)	0.0070 (10)	0.0092 (10)	0.0053 (10)
C14	0.0224 (11)	0.0371 (13)	0.0419 (17)	−0.0015 (10)	0.0019 (9)	0.0095 (11)
C15	0.0265 (12)	0.0303 (12)	0.0308 (11)	−0.0036 (9)	−0.0024 (10)	0.0024 (10)
Sb1	0.02040 (10)	0.02040 (10)	0.01656 (12)	0.0000	0.0000	0.0000
F1	0.0408 (7)	0.0227 (6)	0.0433 (8)	−0.0020 (5)	0.0021 (9)	−0.0044 (8)
F2	0.0568 (16)	0.0371 (13)	0.0171 (9)	0.0069 (13)	0.0000	0.0000

### Tetrapyridinesilver(I) hexafluoroantimonate (II-100K) Geometric parameters (Å, º)

**Table:**

Ag1—N1^i^	2.305 (2)	C13—H131	0.934
Ag1—N1^ii^	2.305 (2)	C14—C15	1.385 (4)
Ag1—N1^iii^	2.305 (2)	C14—H141	0.931
Ag1—N1	2.305 (2)	C15—H151	0.934
N1—C11	1.338 (3)	Sb1—F2^iv^	1.8809 (17)
N1—C15	1.348 (3)	Sb1—F1^v^	1.8776 (13)
C11—C12	1.386 (4)	Sb1—F1^iv^	1.8776 (13)
C11—H111	0.929	Sb1—F1^vi^	1.8776 (13)
C12—C13	1.383 (4)	Sb1—F1	1.8776 (13)
C12—H121	0.929	Sb1—F2	1.8809 (17)
C13—C14	1.384 (4)		
			
N1^i^—Ag1—N1^ii^	107.70 (5)	C15—C14—H141	120.4
N1^i^—Ag1—N1^iii^	107.70 (5)	C14—C15—N1	123.1 (3)
N1^ii^—Ag1—N1^iii^	113.08 (11)	C14—C15—H151	118.6
N1^i^—Ag1—N1	113.08 (11)	N1—C15—H151	118.3
N1^ii^—Ag1—N1	107.70 (5)	F2^iv^—Sb1—F1^v^	90.62 (7)
N1^iii^—Ag1—N1	107.70 (5)	F2^iv^—Sb1—F1^iv^	90.62 (7)
Ag1—N1—C11	121.63 (17)	F1^v^—Sb1—F1^iv^	178.75 (14)
Ag1—N1—C15	120.93 (17)	F2^iv^—Sb1—F1^vi^	89.38 (7)
C11—N1—C15	117.1 (2)	F1^v^—Sb1—F1^vi^	90.01
N1—C11—C12	123.3 (2)	F1^iv^—Sb1—F1^vi^	90.01
N1—C11—H111	118.1	F2^iv^—Sb1—F1	89.38 (7)
C12—C11—H111	118.6	F1^v^—Sb1—F1	90.01
C11—C12—C13	118.9 (3)	F1^iv^—Sb1—F1	90.01
C11—C12—H121	120.7	F1^vi^—Sb1—F1	178.75 (14)
C13—C12—H121	120.4	F2^iv^—Sb1—F2	179.99
C12—C13—C14	118.5 (2)	F1^v^—Sb1—F2	89.38 (7)
C12—C13—H131	120.7	F1^iv^—Sb1—F2	89.38 (7)
C14—C13—H131	120.8	F1^vi^—Sb1—F2	90.62 (7)
C13—C14—C15	119.0 (2)	F1—Sb1—F2	90.62 (7)
C13—C14—H141	120.6		

Symmetry codes: (i) −*x*+1, −*y*+1, *z*; (ii) *y*, −*x*+1, −*z*+1; (iii) −*y*+1, *x*, −*z*+1; (iv) *y*−1/2, −*x*+1/2, −*z*+1/2; (v) −*y*+1/2, *x*+1/2, −*z*+1/2; (vi) −*x*, −*y*+1, *z*.
